# Supplementary material for: Proenkephalin deletion in hematopoietic cells induces intestinal barrier failure resulting in clinical feature similarities with irritable bowel syndrome in mice
Source: Commun Biol. 2023 Nov 16;6:1168. doi: 10.1038/s42003-023-05542-2 (PMC10652007; doi:10.1038/s42003-023-05542-2)
Supplement: Supplementary file 1 — Reporting Summary [file 42003_2023_5542_MOESM1_ESM.pdf]

nature portfolio

Corresponding author(s): Dietrich Gilles

Last updated by author(s): Oct 5, 2023

Reporting Summary

Nature Portfolio wishes to improve the reproducibility of the work that we publish. This form provides structure for consistency and transparency in reporting. For further information on Nature Portfolio policies, see our Editorial Policies and the Editorial Policy Checklist.

Statistics

For all statistical analyses, confirm that the following items are present in the figure legend, table legend, main text, or Methods section.

n/a	Confirmed	
☐	☒	The exact sample size (*n*) for each experimental group/condition, given as a discrete number and unit of measurement
☐	☒	A statement on whether measurements were taken from distinct samples or whether the same sample was measured repeatedly
☐	☒	The statistical test(s) used AND whether they are one- or two-sided<br>*Only common tests should be described solely by name; describe more complex techniques in the Methods section.*
☒	☐	A description of all covariates tested
☐	☒	A description of any assumptions or corrections, such as tests of normality and adjustment for multiple comparisons
☐	☒	A full description of the statistical parameters including central tendency (e.g. means) or other basic estimates (e.g. regression coefficient) AND variation (e.g. standard deviation) or associated estimates of uncertainty (e.g. confidence intervals)
☒	☐	For null hypothesis testing, the test statistic (e.g. *F*, *t*, *r*) with confidence intervals, effect sizes, degrees of freedom and *P* value noted<br>*Give P values as exact values whenever suitable.*
☒	☐	For Bayesian analysis, information on the choice of priors and Markov chain Monte Carlo settings
☒	☐	For hierarchical and complex designs, identification of the appropriate level for tests and full reporting of outcomes
☒	☐	Estimates of effect sizes (e.g. Cohen's *d*, Pearson's *r*), indicating how they were calculated

Our web collection on statistics for biologists contains articles on many of the points above.

Software and code

Policy information about availability of computer code

Data collection	FlowJo_v10
Data analysis	GraphPad_v9, R studio, FlowJo_v10 - Plugin UMAP v3.1 & phenograph v2.4

For manuscripts utilizing custom algorithms or software that are central to the research but not yet described in published literature, software must be made available to editors and reviewers. We strongly encourage code deposition in a community repository (e.g. GitHub). See the Nature Portfolio guidelines for submitting code & software for further information.

Data

Policy information about availability of data

All manuscripts must include a data availability statement. This statement should provide the following information, where applicable:

- Accession codes, unique identifiers, or web links for publicly available datasets
- A description of any restrictions on data availability
- For clinical datasets or third party data, please ensure that the statement adheres to our policy

All data are available as supplementary materials. All other data are available from the corresponding author on reasonable request.

Research involving human participants, their data, or biological material

Policy information about studies with human participants or human data. See also policy information about sex, gender (identity/presentation), and sexual orientation and race, ethnicity and racism.

Reporting on sex and gender	*Use the terms sex (biological attribute) and gender (shaped by social and cultural circumstances) carefully in order to avoid confusing both terms. Indicate if findings apply to only one sex or gender; describe whether sex and gender were considered in study design; whether sex and/or gender was determined based on self-reporting or assigned and methods used. Provide in the source data disaggregated sex and gender data, where this information has been collected, and if consent has been obtained for sharing of individual-level data; provide overall numbers in this Reporting Summary. Please state if this information has not been collected. Report sex- and gender-based analyses where performed, justify reasons for lack of sex- and gender-based analysis.*
Reporting on race, ethnicity, or other socially relevant groupings	*Please specify the socially constructed or socially relevant categorization variable(s) used in your manuscript and explain why they were used. Please note that such variables should not be used as proxies for other socially constructed/relevant variables (for example, race or ethnicity should not be used as a proxy for socioeconomic status). Provide clear definitions of the relevant terms used, how they were provided (by the participants/respondents, the researchers, or third parties), and the method(s) used to classify people into the different categories (e.g. self-report, census or administrative data, social media data, etc.) Please provide details about how you controlled for confounding variables in your analyses.*
Population characteristics	*Describe the covariate-relevant population characteristics of the human research participants (e.g. age, genotypic information, past and current diagnosis and treatment categories). If you filled out the behavioural & social sciences study design questions and have nothing to add here, write "See above."*
Recruitment	*Describe how participants were recruited. Outline any potential self-selection bias or other biases that may be present and how these are likely to impact results.*
Ethics oversight	*Identify the organization(s) that approved the study protocol.*

Note that full information on the approval of the study protocol must also be provided in the manuscript.

Field-specific reporting

Please select the one below that is the best fit for your research. If you are not sure, read the appropriate sections before making your selection.

☒ Life sciences ☐ Behavioural & social sciences ☐ Ecological, evolutionary & environmental sciences

For a reference copy of the document with all sections, see nature.com/documents/nr-reporting-summary-flat.pdf

Life sciences study design

All studies must disclose on these points even when the disclosure is negative.

Sample size	The experimental design was optimized and adjusted to minimize animal use while still achieving both the scientific objectives and statistical analyses. Sample size was at least 5 mice per group.
Data exclusions	No data were excluded
Replication	The data have been obtained from 6 sets of experiments. Each set of experiments included at least 5 chimeric mice per group. The overall study required a large number of animals since most of them could not be performed on the same set of experiments.The results were thus obtained and/or compilated from mice originating from different sets of experiments.
Randomization	Hematopoietic cells were prepared from PENK+/+ and PENK-/- mice. Recipient mice were then blindly assigned.
Blinding	Collaborators from external laboratories who performed experiments were not informed on the mice. The identification of the chimeras was revealed at the end of each experimentation for statistical analyses. When experiments were realized in our laboratory, mouse assignments were done by an animal caretaker not involved in the study.

Reporting for specific materials, systems and methods

We require information from authors about some types of materials, experimental systems and methods used in many studies. Here, indicate whether each material, system or method listed is relevant to your study. If you are not sure if a list item applies to your research, read the appropriate section before selecting a response.

Materials & experimental systems

n/a	Involved in the study
☐	☒ Antibodies
☒	☐ Eukaryotic cell lines
☒	☐ Palaeontology and archaeology
☐	☒ Animals and other organisms
☒	☐ Clinical data
☒	☐ Dual use research of concern
☒	☐ Plants

Methods

n/a	Involved in the study
☒	☐ ChIP-seq
☐	☒ Flow cytometry
☒	☐ MRI-based neuroimaging

Antibodies

Antibodies used	All antibodies are listed in a table
Validation	All the antibodies used in the experiments were QC tested by the manufacturer and titrated by ourselves. Background staining was examined by using isotype matched control Ig.

Animals and other research organisms

Policy information about studies involving animals; ARRIVE guidelines recommended for reporting animal research, and Sex and Gender in Research

Laboratory animals	Species, strain and ages of the animals are reported in the corresponding section of materials and methods
Wild animals	The study did not involved wild animals
Reporting on sex	Findings apply to only male mice.<br>We used male only to avoid the confounding effects of sex hormones on experimental outcomes.
Field-collected samples	No field-collected samples were used.
Ethics oversight	Animal Care and Ethics Committee of US006/CREFRE (CEEA-122; application number APAFIS #16385-2018080222083660 v3)

Note that full information on the approval of the study protocol must also be provided in the manuscript.

Flow Cytometry

Plots

Confirm that:

☒ The axis labels state the marker and fluorochrome used (e.g. CD4-FITC).

☒ The axis scales are clearly visible. Include numbers along axes only for bottom left plot of group (a 'group' is an analysis of identical markers).

☒ All plots are contour plots with outliers or pseudocolor plots.

☒ A numerical value for number of cells or percentage (with statistics) is provided.

Methodology

Sample preparation	Spleen, Peyer's Patches, and colonic cells were collected, prepared and analysed as described in the materials and methods section
Instrument	BD LSR Fortessa X20 and BD Symphony
Software	Diva software has been used for acquisition, FlowJo for the analyses, and GraphPad-prism for statistical analyses
Cell population abundance	Reported in the corresponding materials and methods section and in the main text when required
Gating strategy	Gating strategies are included in the figures

☒ Tick this box to confirm that a figure exemplifying the gating strategy is provided in the Supplementary Information.
