# Supplementary material for: Proenkephalin deletion in hematopoietic cells induces intestinal barrier failure resulting in clinical feature similarities with irritable bowel syndrome in mice
Source: Commun Biol. 2023 Nov 16;6:1168. doi: 10.1038/s42003-023-05542-2 (PMC10652007; doi:10.1038/s42003-023-05542-2)
Supplement: Supplementary file 2 — Supplementary Information [file 42003_2023_5542_MOESM2_ESM.pdf]

Proenkephalin deletion in hematopoietic cells induces intestinal barrier failure resulting in clinical feature similarities with irritable bowel syndrome in mice

Xavier Mas-Orea[1#], Lea Rey[1#], Louise Battut[1], Cyrielle Bories[2], Camille Petitfils[1], Anne Abot[1##], Nadine Gheziel[1,2], Eve Wemelle[1], Catherine Blanpied[1], Jean-Paul Motta[1], Claude Knauf[1], Frederick Barreau[1], Eric Espinosa[1], Meryem Aloulou[2], Nicolas Cenac[1], Matteo Serino[1], Lionel Mouledous[3], Nicolas Fazilleau[2], and Gilles Dietrich[1*]

[1] IRSD, Université de Toulouse, INSERM, INRAE, ENVT, Univ Toulouse III - Paul Sabatier (UPS), Toulouse, France

[2]INFINITy, Université de Toulouse, INSERM U1291, CNRS U5051, Univ Toulouse III - Paul Sabatier (UPS), Toulouse, France

[3]Research Center on Animal Cognition (CRCA), Center of Integrative Biology (CBI), Université de Toulouse, CNRS UMR-5169, Univ Toulouse III - Paul Sabatier (UPS), Toulouse, France.

[#] These authors contributed equally

[##]Present address: Enterosys SAS, Labège, France.

[*]**Correspondence**: Gilles Dietrich, Digestive Health Research Institute (IRSD), CHU Purpan BP 3028, 31024 Toulouse Cedex 3, France. Tel.: +33 5 62 74 45 02; fax: +33 62 74 45 58; e-mail: gilles.dietrich@inserm.fr

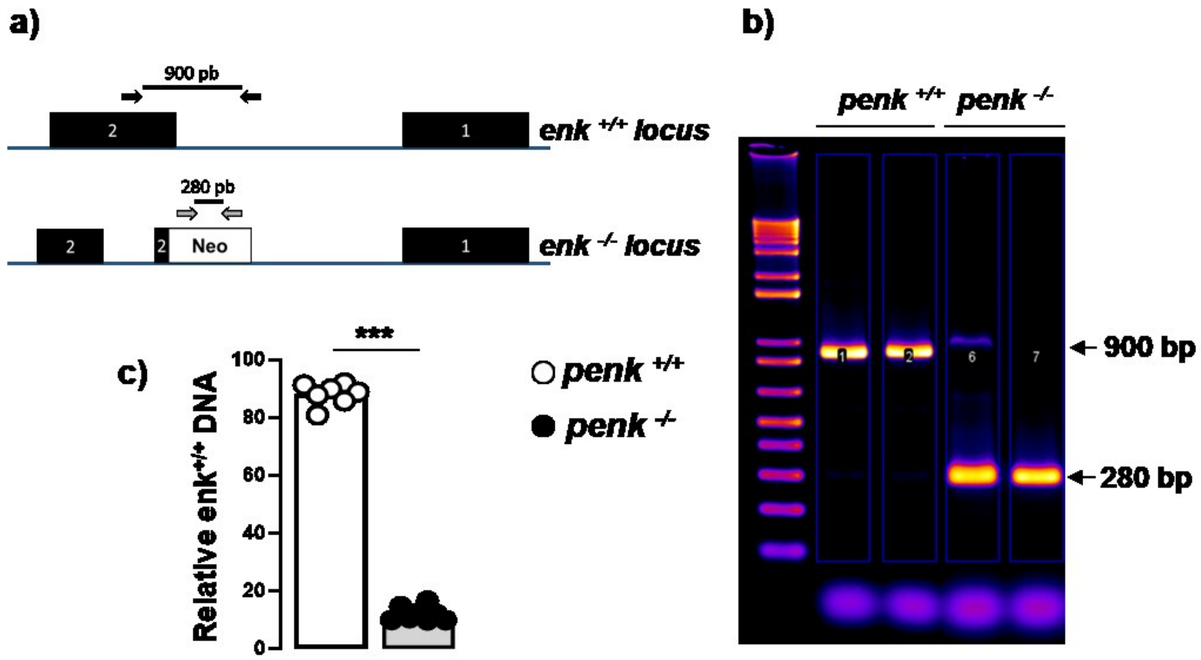

Supplementary figure 1. Evaluation of the chimerism. Hematopoietic chimerism was assessed by quantifying donor *enk* DNA in the recipient's bone marrow. Engraftment status was examined by PCR using primers amplifying either *enk* locus (*penk*$^{+/+}$) (900 bp) or neomycin box inserted into exon 2 (*penk*$^{-/-}$) (280 bp) [1] (**a**). As exemplified in (**b**) for 2 *penk*$^{+/+}$ and 2 *penk*$^{-/-}$ chimeras, PCR products stained with GelRed® were quantified by measuring fluorescence intensity (FI) with Fiji/ImageJ software. The chimerism level was calculated as follows: FI enk gene amplicon (900 bp) / (FI enk gene amplicon (900 bp) + FI Neomycin gene amplicon (280 bp)). The *enk* gene locus represented less than 8 % in bone marrow cells from *penk*$^{-/-}$ engrafted recipients (n = 7) (**c**).

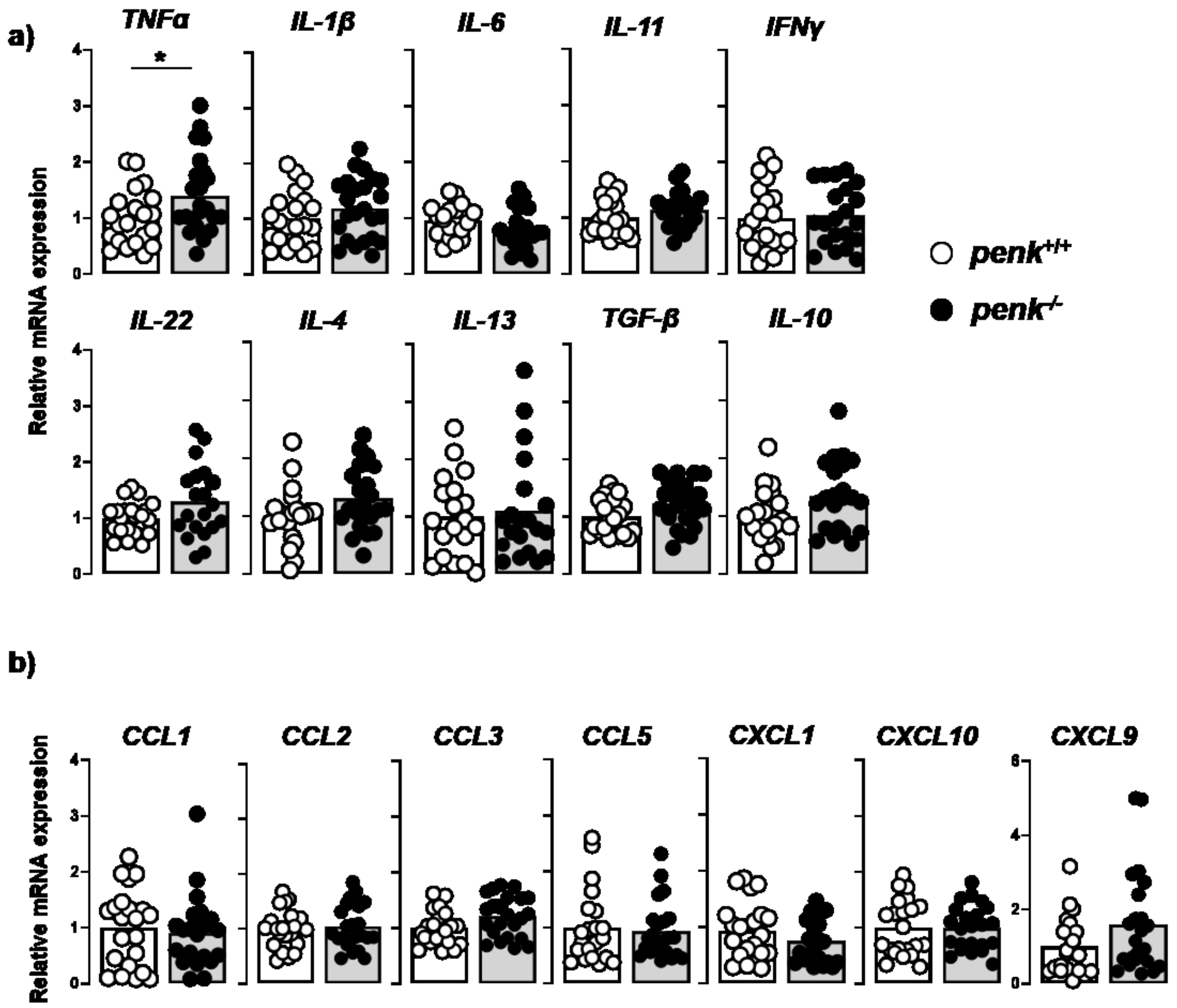

Supplementary figure 2. *Penk*-deficient chimeric mice do not exhibit alteration of the cytokine and chemokine mRNA expression profiles in the gut. $penk^{+/+}$ (white circles and white histogram) and $penk^{-/-}$ (black circles and grey histogram) chimeric mice were assessed for mRNA expression levels of a number of both cytokines (**a**) and chemokines (**b**). mRNA encoding for the cytokines TNFα, IL-1β, IL-6, IL-11, IFNγ, IL-22, Il-4, IL-13, TGF-β and IL-10 and the chemokines CCL1, CCL2, CCL3, CCL5, CXCL1, CXCL10, CXCL9 was quantified by real-time qPCR in colonic biopsies using specific forward and reverse primers (**Supplementary table 2**). The mRNA content was normalized to that of the HPRT. The $2^{-\Delta\Delta CT}$ method was used to evaluate mRNA expression levels in $penk^{-/-}$ chimeric mice relative to the

penk$^{+/+}$ control chimeric mice. Each symbol represents 1 mouse (n = 19 to 24). Statistical analysis was performed using Mann-Whitney U test.

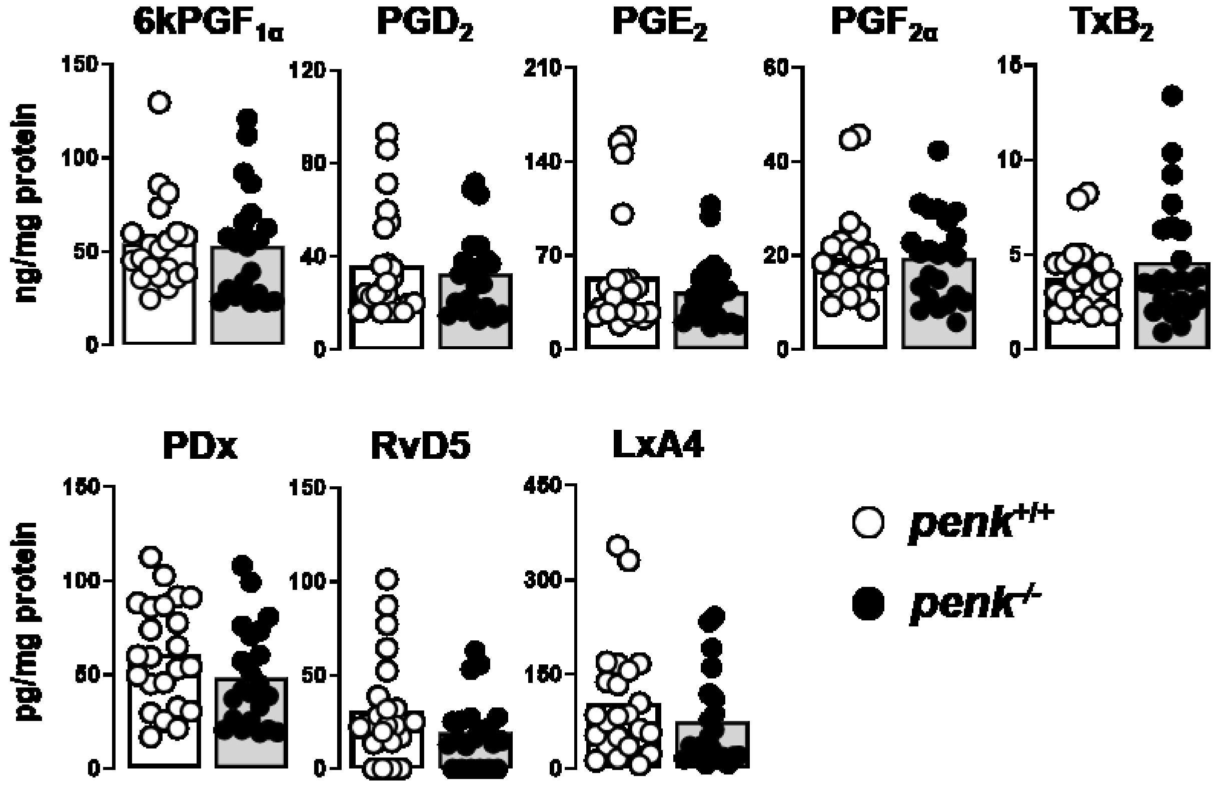

Supplementary figure 3. ***Penk*-deficient chimeric mice do not exhibit alteration of the pro-inflammatory and pro-resolving bioactive lipids in the gut.** Pro-inflammatory (upper panels) and pro-resolving (lower panels) bioactive lipids were quantified by liquid chromatography coupled to tandem mass spectrometry (LC-MS/MS) as expressed in pg or ng per milligrams of colonic tissue proteins. Each symbol represents 1 mouse (n = 23). Statistical analysis was performed using Mann-Whitney U test.

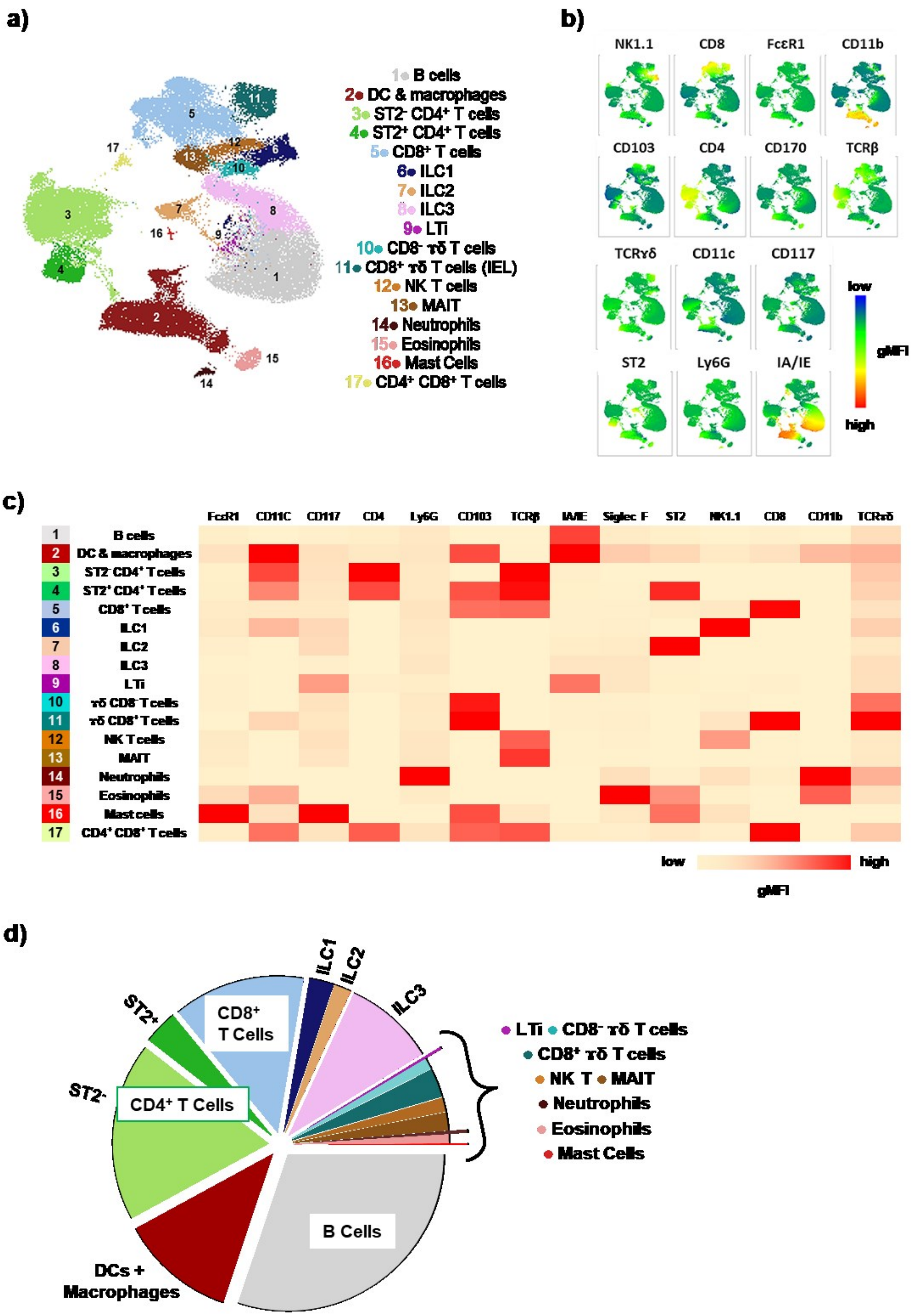
a)
1 B cells
2 DC & macrophages
3 ST2- CD4+ T cells
4 ST2+ CD4+ T cells
5 CD8+ T cells
6 ILC1
7 ILC2
8 ILC3
9 LTi
10 CD8- τδ T cells
11 CD8+ τδ T cells (IEL)
12 NK T cells
13 MAIT
14 Neutrophils
15 Eosinophils
16 Mast Cells
17 CD4+ CD8+ T cells
b)
NK1.1
CD8
FcεR1
CD11b
CD103
CD4
CD170
TCRβ
TCRγδ
CD11c
CD117
ST2
Ly6G
IA/IE
low
gMFI
high
c)
FcεR1
CD11C
CD117
CD4
Ly6G
CD103
TCRβ
IA/IE
Siglec F
ST2
NK1.1
CD8
CD11b
TCRτδ
1 B cells
2 DC & macrophages
3 ST2- CD4+ T cells
4 ST2+ CD4+ T cells
5 CD8+ T cells
6 ILC1
7 ILC2
8 ILC3
9 LTi
10 τδ CD8- T cells
11 τδ CD8+ T cells
12 NK T cells
13 MAIT
14 Neutrophils
15 Eosinophils
16 Mast cells
17 CD4+ CD8+ T cells
low
high
gMFI
d)
ILC1
ILC2
ILC3
CD8+ T Cells
ST2+
ST2-
CD4+ T Cells
LTi
CD8- τδ T cells
CD8+ τδ T cells
NK T
MAIT
Neutrophils
Eosinophils
Mast Cells
B Cells
DCs + Macrophages

Supplementary figure 4. Visualization of flux cytometry data using self-organizing map clustering. a) UMAP projection of the $CD45^+$ cell phenotyping showing every clusters defined by phenograph**. b**) Relative density of each cell surface marker within the self-organizing maps generated by using the Uniform Manifold Approximation and Projection (UMAP) method. **c**) Heatmap of fluorescent signal expression (gMFI) for the indicated markers across the Phenograph clusters. **d**) Relative distribution of immune cell subsets in the colon mucosa of *penk*$^{+/+}$ chimeric mice (n = 11).

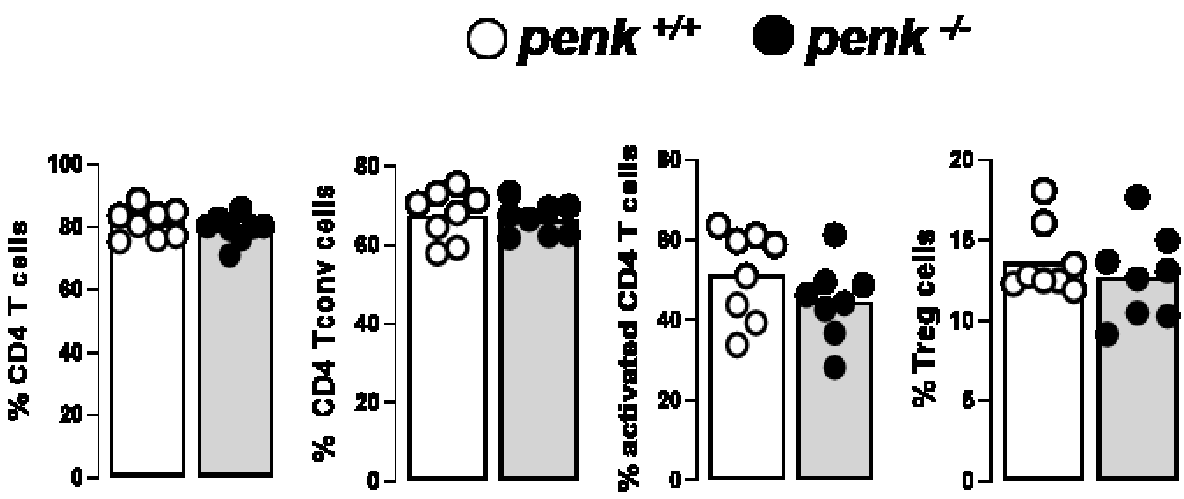

Supplementary figure 5. ***Penk*-deficient chimeric mice display no functional CD4$^+$ T cell alterations in Peyer's patches.** Frequency of CD4$^+$ T lymphocytes, conventional CD4$^+$ T lymphocytes, activated conventional CD4$^+$ T lymphocytes and regulatory T cells among living CD45$^+$ TCRβ-expressing cells in Peyer's patches from *penk*$^{+/+}$ (white circles and white histogram) and *penk*$^{-/-}$ (black circles and grey histogram) chimeric mice (n = 8) as assessed by cytofluorometry. Each symbol represents 1 mouse. Statistical analysis was performed using Mann-Whitney U test.

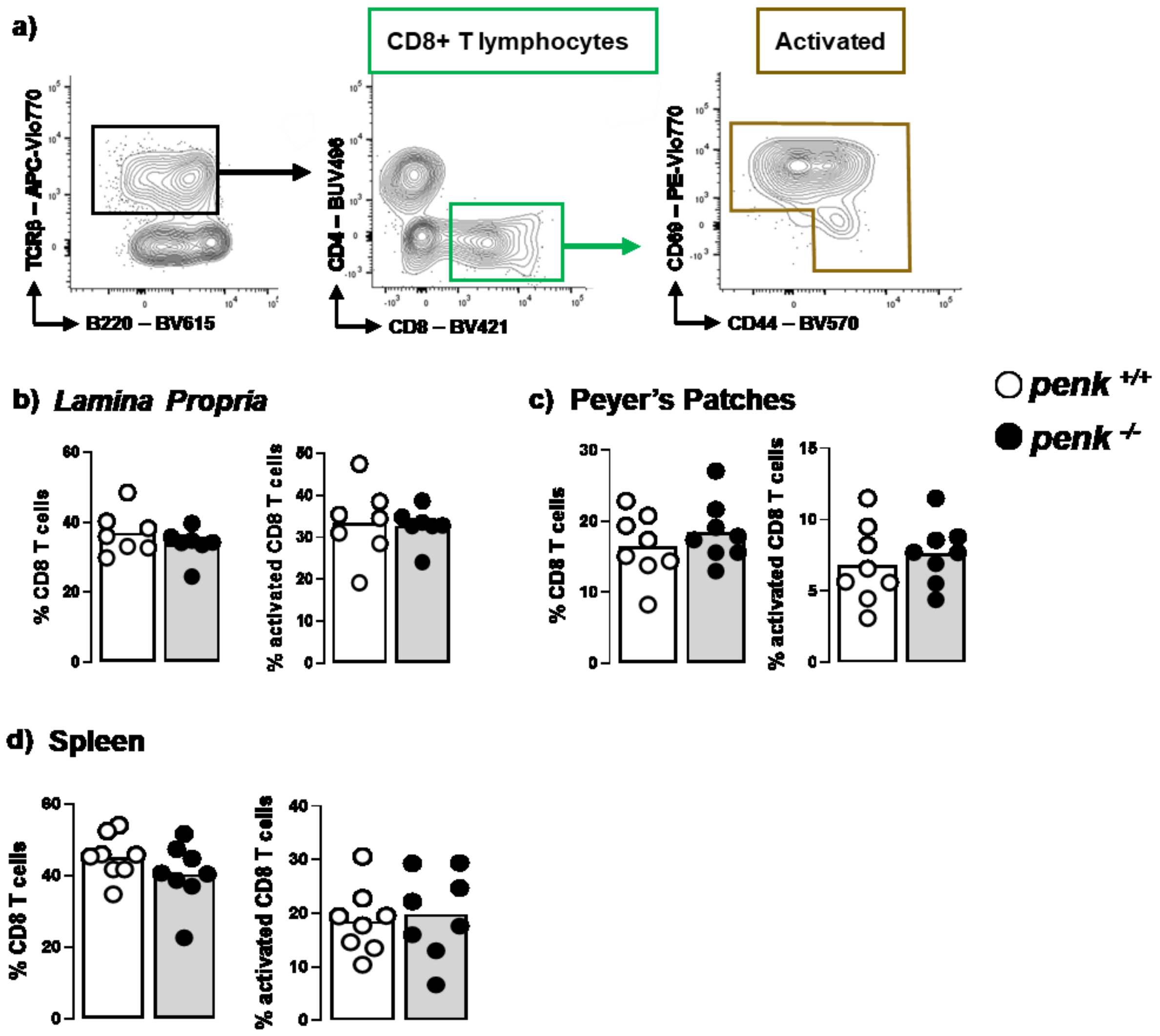

Supplementary figure 6. ***Penk*-deficient chimeric mice display no functional CD8$^+$ T cell alteration.** The relative frequency of CD8$^+$ T lymphocyte subsets in colonic *lamina propria*, Peyer's patches and spleen from *penk*$^{+/+}$ (white circles and white histogram) and *penk*$^{-/-}$ (black circles and grey histogram) chimeric mice was estimated by cytofluorometry. **a**) Gating strategy for flow cytometry data analysis. Living CD45-expressing cells gated on TCRβ chain$^+$ CD8$^+$ T lymphocytes were defined as activated based on the expression of CD44 and/or CD69 cell surface markers. **b - d**) Frequency of total CD8$^+$ T lymphocytes and activated CD8$^+$ T lymphocytes among living CD45$^+$ TCRβ-expressing cells in the *lamina propria* (n = 7) (**b**), Peyer's patches (n = 8) (**c**) and spleen (n = 8) (**d**). Each symbol represents 1 mouse. Statistical analysis was performed using Mann-Whitney U test.

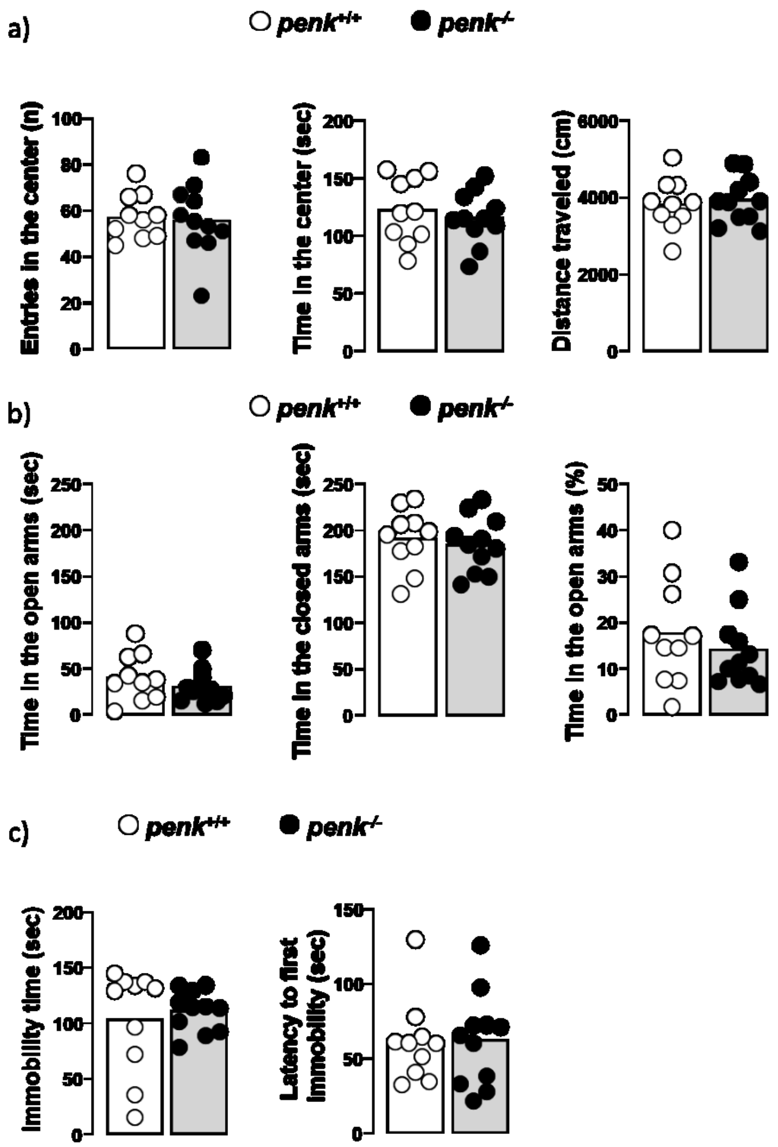

Supplementary figure 7. Behavioral assessment of mice. In addition to the novelty suppressed feeding test, *penk*$^{+/+}$ (white circles and white histogram) and *penk*$^{-/-}$ (black circles and grey histogram) chimeric mice were analyzed using three other behavioral tests including open field (**a**), elevated plus maze (**b**) and tail suspension (**c**).

a) Number of entries (left panel) and time spent in the center of the open field (middle panel). Total ambulatory distance (right panel) was calculated to ensure the absence of locomotor alteration. **b**) Time spent in the open arms (left panel) and the closed arms (middle panel) of the elevated plus maze. The relative number of entries in the open arms is shown in the right panel.

c) Immobility time (left panel) and latency to the first immobility (right panel) recorded in the tail suspension test. Each symbol represents 1 mouse (n = 10 *versus* 11). Statistical analysis was performed using Mann-Whitney U test. No significant difference was observed.

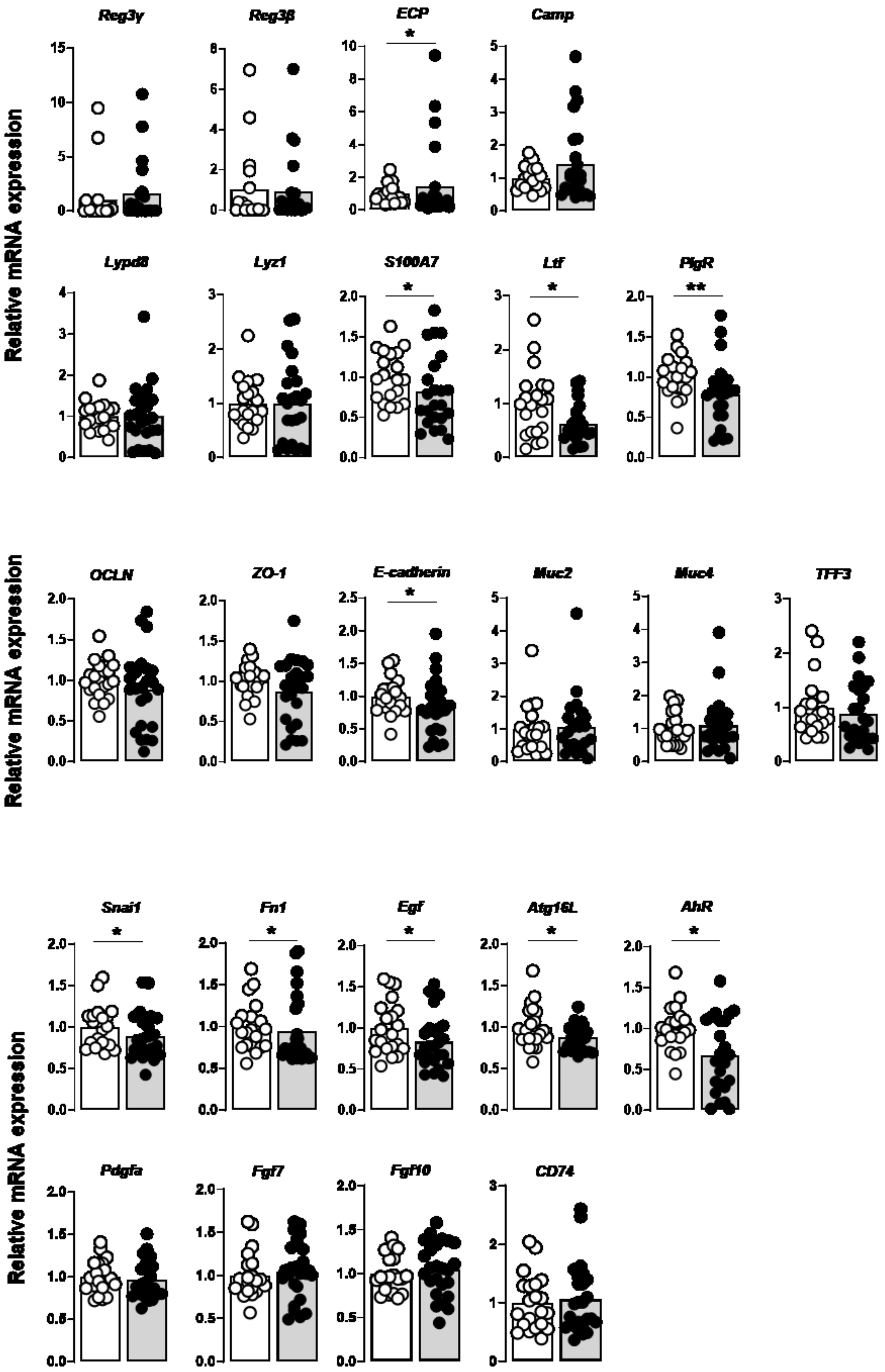
Reg3γ
Reg3β
ECP
Camp
Lypd8
Lyz1
S100A7
Ltf
PIgR
Relative mRNA expression
OCLN
ZO-1
E-cadherin
Muc2
Muc4
TFF3
Relative mRNA expression
Snai1
Fn1
Egf
Atg16L
AhR
Pdgfa
Fgf7
Fgf10
CD74
Relative mRNA expression

Supplementary figure 8. *Penk*-deficient chimeric mice exhibit decreased levels of mRNA coding antimicrobial peptides and pro-repair/regenerative mediators in the gut. *penk*$^{+/+}$ (white circles and white histogram) and *penk*$^{-/-}$ (black circles and grey histogram) chimeric mice were assessed for mRNA expression levels of a number of mediators involved in epithelial homeostasis including repair/regenerative mediators (**a**), junctional complex proteins, mucins (**b**) and antimicrobial peptides (**c**). mRNAs were quantified by real-time qPCR in colonic biopsies using specific forward and reverse primers (**Supplementary table 2**). The mRNA content was normalized to that of the HPRT. The $2^{-\Delta\Delta CT}$ method was used to evaluate mRNA expression levels in *penk*$^{-/-}$ chimeric mice relative to the *penk*$^{+/+}$ chimeric mice. Each symbol represents 1 mouse (n = 19 *versus* 24). Statistical analysis was performed using Mann-Whitney U test.

Reg3γ, *Reg3β* (regenerating islet-derived genes); *ECP* (eosinophil cationic protein); *Camp* (cathelicidin antimicrobial peptide); *Lypd8* (LY6/PLAUR domain containing 8); *Lyz1* (lyzozyme 1); *S100A7* (psoriasin); *Ltf* (lactoferin); *PIgR* (polymeric immunoglobulin receptor); *OCLN* (occluding); *TJP1* (tight junction protein 1, zona occludens 1); CDH1 (cadherin 1, E-cadherin); Muc2, Muc4 (mucin, oligomeric mucus/gel-forming); TFF3 (trefoil factor 3); Snai1 (snail family transcriptional repressor 1); Fn1 (fibronectin 1); Egf (epidermal growth factor); Atg16L1 (autophagy related 16 like 1); AhR (aryl hydrocarbon receptor); Pdgfa (platelet-derived growth factor subunit A); Fgf7, Fgf10 (fibroblast growth factor); CD74 (a receptor for the macrophage migration inhibitory factor (MIF) on many cell types).

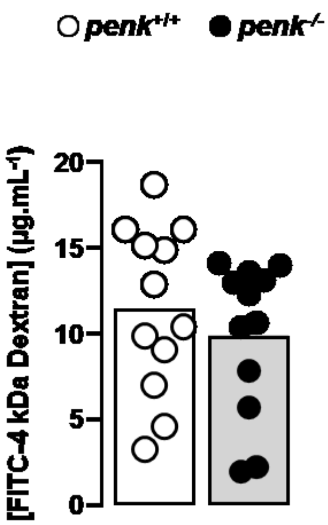

Supplementary figure 9. Peripheral opioid receptor blockade by naloxone-methiodide does not alter paracellular intestinal permeability in non-irradiated mice. Male C57BL/6JRj mice were intraperitoneally injected with 200 µL of either PBS (white circles and white histogram) or 10 mg mL^{-1} naloxone-methiodide (Sigma Chemical Co., St Louis, MO) (2 mg/mouse) two days apart (black circles and grey histogram) [2]. On day 7 (one day after the fourth injection) *in vivo* measurement of the paracellular permeability expressed as serum 4 kDa FITC-dextran concentration was determined 4 hours after gavage. Each symbol represents 1 mouse (n = 12). Statistical analysis was performed using Mann-Whitney U test.

Supplementary table 1. Experimental design.

Sets of experiments	1	2a	2b	3	4	5
Colorectal distension	X					
Gut transit assessment		X				
Behavioral testing						X
Intestinal permeability				*in / ex vivo*	*in vivo*	
Colonic tissue analysis		X				X
Colon contraction assessment			X			
Biofilm analysis (Colon)				X		X
Colon inflammatory parameters			X	X		X
Cytometry analysis (LP, PP, spleen)	X				X	
Microbiota taxonomy and IgA-coated bacteria					X	
Colon RNA sequencing		X				
Penk content in immune cells		X				

To carry out all the experiments reported in the study, five series (numbered from 1 to 5) of hematopoietic *penk*$^{+/+}$ and *penk*$^{-/-}$ chimeric mice were generated in different periods of time (the third generation of chimeras were divided in two groups). Chimerism was quantified on bone marrow cells from mice belonging to the first set of chimeras (**Supplementary figure 1**) and then randomly assessed (but not quantified) in few mice from the other sets of experiments. *In vivo* experiments are indicated in blue. Colonic tissue analysis includes macroscopic and histological assessments as well as apoptosis quantification, inflammation parameters include all RT-qPCR and quantification of bioactive lipids, cytometry analysis was performed on colonic *lamina propria* (LP), Peyer's patches (PP) and spleen. Intestinal

permeability *ex vivo* was performed on ileum, Peyer's patches and colonic biopsies. The number of animals used for each specific protocol is indicated in the figure legends.

Supplementary table 2. Primers used for qPCR.

Transcript	Forward	Reverse
Hprt	GTTCTTTGCTGACCTGCTGGAT	CCCCGTTGACTGATCATTACAG
Tnfa	CCACGCTCTTCTGTCTACTGAAC	GGTCTGGGCCATAGAACTGATG
Il1b	ACCTTCCAGGATGAGGACATGAG	CATCCCATGAGTCACAGAGGATG
Il6	TCTGGGAAATCGTGGAAATGAG	TTCTGCAAGTGCATCATCGTTG
Il11	GGATCTTTGCAGCTTCCTGG	TGCCGGAGGTAGGACATCA
Ifng	CAGCAACAGCAAGGCGAAA	AGCTCATTGAATGCTTGGCG
Il22	ACCGCTGATGTGACAGGAGC	AGGTGGTGCCTTTCCTGACC
Il4	GGCTTTTCGATGCCTGGATT	CTTGGACTCATTCATGGTGCAG
Il13	GGCAGCATGGTATGGAGTGTG	GCAATTGGAGATGTTGGTCAGG
Tgfb	GACCCCCACTGATACGCCT	GCTGAATCGAAAGCCCTGTA
Il10	ATTTGAATTCCCTGGGTGAGAAG	CACAGGGGAGAAATCGATGACA
Cxcl1	GCTAAAAGGTGTCCCCAAGTAACG	TCACCAGACAGGTGCCATCA
Cxcl10	GGGTCTGAGTGGGACTCAAGG	TCGCAGGGATGATTTCAAGC
Cxcl9	AGTGTGGAGTTCGAGGAACCC	CTGTTTGAGGTCTTTGAGGGATTT
Ccl1	TGATCCCCCAGCTGTGGTAT	TGATTTTGAACCCACGTTTTGT
Ccl2	ACGTGTTGGCTCAGCCAGAT	TGATCCTCTTGTAGCTCTCCAGC
Ccl3	CCAAGTCTTCTCAGCGCCATAT	CAAAGGCTGCTGGTTTCAAAATA
Ccl5	ACTCCCTGCTGCTTTGCCTAC	TTCCTTCGAGTGACAAACACGA
penk	CGACATCAATTTCCTGGCGT	AGATCCTTGCAGGTCTCCCA
AhR	CGCTTGATTTACAGAAATGGA	ATCTCGTACAACACAGCCTC
Snai1	TGTCTGCACGACCTGTGGAAAG	AGCAGGAGAATGGCTTCTCAC
Fn1	TGGTGGCCACTAAATACGAA	GGAGGGCTAACATTCTCCAG
Fgf7	TGTTCTGTCGCACCCAGTGGTA	TTCCAACTGCCACGGTCCTGAT
Fgf10	CCTTCACCAAGTACTTTCTCAC	CGGCAACAACTCCGATTTCCAC
Egf	ACTGGTGTGACACCAAGAGGTC	CCACAGGTGATCCTCAAACACG
Pdgfa	CTGGCTCGAAGTCAGATCCACA	GACTTGTCTCCAAGGCATCCTC
Cd74	GCTGGATGAAGCAGTGGCTCTT	GATGTGGCTGACTTCTTCCTGG
Atg16l	GGACACTCATCCTGCTTCTGGT	GCTTCCCAAAGTTTCACCCTGC
Occludin	TGGATGACTACAGAGAGGAGAGT	TCCTCTTGATGTGCGATAATTTGC
ZO-1=Tjp1	GTTGGTACGGTGCCCTGAAAGA	GCTGACAGGTAGGACAGACGAT
E-cadherin	CCAATCCTGATGAAATTGGAAACT	AACACCAACAGAGAGTCGTAAG
Muc2	CGGAACTCCAGAAAGAAGCCA	GGCAGTCAGACGCAAAGTTGTA
Muc4	AGAGGCAGAAGAGGAGTGGAGA	GGTGGTAGCCTTTGTAGCCATC

Reg3γ	CCTCCATGATCAAAAGCAGTGG	GGATTCGTCTCCCAGTTGATGT
Reg3β	TGGCTCCTACTGCTATGCCTTG	CGCTATTGAGCACAGATACGAGG
Ecp	CATCACCAGTCGGAGGAGAACA	ATGGGACTGTCCTGTGGAGTTC
Camp	CTTCAACCAGCAGTCCCTAGAC	GCCACATACAGTCTCCTTCACTC
Lypd8	AACACTCTGCGAGGAGAAACCC	AGAACAGCCCTTCAGCTCCACT
Lyz1	ATGGCTACCGTGGTGTCAAG	CGGTCTCCACGGTTGTAGTT
S100A7	GATAGTGTGCCTCGCTTCATGG	CTGGAGATGGTAGTCCTTCACC
Tff3	TGCAGATTACGTTGGCCTGTC	TGGAGTCAAAGCAGCAGCC
Ltf	GTCTGCCATTGGCTTTGTGAGG	CCTTTGAGGCTATCACATCCTGC
PIgR	AGCAAGGCCAGACCTATGGAG	GGTTGACATGGGATGACCCT

Supplementary table 3. Antibodies used for cytofluorometry analysis

Target antigen	Antibody clone	Fluorochrome	Provider
CD45	REA737	VioGreen	Miltenyi
TCRβ	H57-597	BV711	BioLegend
CD8α	REA601	PE-Vio 770	Miltenyi
TCRγδ	REA633	PerCP-Vio 700	Miltenyi
CD11b	REA592	PE-Vio 615	Miltenyi
FcεR1	REA1079	APC	Miltenyi
Ly6G	REA526	VioBlue	Miltenyi
CD117	2B8	BUV395	BD Biosciences
CD103	2E7	BV650	BD Biosciences
ST2	U29-93	PE	BD Biosciences
CD170 (SiglecF)	E50-2440	Alexa Fluor 488	BD Biosciences
CD4	GK1.5	BUV737	BD Biosciences
NK1.1	S17016D	PE-Cyanine5	BioLegend
MHC II (IA/IE)	M5/114.15.2	BV785	BioLegend
CD11c	N418	Alexa Fluor 700	BioLegend
TCR-β chain	REA318	APC-Vio 770	Miltenyi
CD4	RM4-5	BUV496	BD Biosciences
CD45	30-F1	PE-CF594	BD Biosciences
CD62L	MEL-14	Alexa Fluor 700	Invitrogen
CD8α	53-6.7	BV421	BD Biosciences
CD44	IM7	BV570	BioLegend
CD11b	M1/70	BUV395	BD Biosciences
B220/CD45R	RA3-6B2	BUV615	BD Biosciences
CD103	REA789	VioBright 515	Miltenyi
CD25 (IL2Rα)	REA568	APC	Miltenyi
CD69	REA937	PE-Vio 770	Miltenyi
FoxP3	REA788	PE	Miltenyi
GL7	GL7	Alexa Fluor 647	BD Biosciences
Fas	Jo2	PE-Cyanine7	BD Biosciences
CD38	90	PE-Cyanine5	ThermoFischer
IgM	II/41	PE	ThermoFischer
CD138	281-2	BV605	BioLegend
CD19	1D3	PE-CF594	BD Biosciences
B220/CD45R	RA3-6B2	APC/Cyanine7	BD Biosciences
IgA	C10-3	FITC	BD Biosciences
IgD	11-26c.2a	BV421	BioLegend
TCRβ	REA318	APCVio770	Miltenyi
CD11b	M1/70	BB515	BD Biosciences
CD4	REA604	VioBlue	Miltenyi
CD8α	REA601	PEVio770	Miltenyi
CD45	REA737	APC	Miltenyi
B220	REA755	PercPVio700	Miltenyi

1 Konig, M. et al. Pain responses, anxiety and aggression in mice deficient in pre-proenkephalin. Nature 383, 535-538, doi:10.1038/383535a0 (1996).

2 Jaume, M., Laffont, S., Chapey, E., Blanpied, C. & Dietrich, G. Opioid receptor blockade increases the number of lymphocytes without altering T cell response in draining lymph nodes in vivo. J Neuroimmunol 188, 95-102, doi:10.1016/j.jneuroim.2007.06.013 (2007).
